# Predictors of unmet need for family planning among all women of reproductive age in Ethiopia

**DOI:** 10.1186/s40834-019-0087-z

**Published:** 2019-06-04

**Authors:** Afework Tadele, Dessie Abebaw, Rahma Ali

**Affiliations:** 1 Population and family health, Jimma University, Jimma, Ethiopia; 2 Epidemiology, Jimma University, Jimma, Ethiopia

**Keywords:** Unmet need, Family planning, Ethiopia, PMA2020

## Abstract

**Introduction:**

Contraception is a good indicator of the extent to which couples have access to reproductive health services. Survey data on unmet need can provide overall direction by helping to pinpoint the obstacles in society and weaknesses in services that need to be overcome. This study is significant as it provides strong policy recommendations for the design and implementation of economic and non-economic interventions into family planning utilization by all eligible women.

**Objective:**

To Identify Predictors of Unmet Need of Family Planning in Ethiopia.

**Methods:**

A national level survey by performance monitoring and accountability (PMA 2020), which conducted between March and April 2016 among 7552 all women of 15–49 years were utilized. Stata® version13 were used for survey data for analysis using weighted frequency to give equal chances for enumeration areas represented. Binary and multivariate logistic regression employed. *P*-value < 0.05 were used to declare independent predictors of unmet need for family planning in Ethiopia.

**Result:**

7494 women responded to the interview giving response rate of 99.2%. Overall unmet need for family planning was 1, 214 (16.2%) of which 772 (10.3%) was for spacing and 450 (6.0%) for limiting. Overall unmet need was 540 (7.2%) in urban and 1431(19.1%) in rural areas of the Ethiopia. Statistically significant predictors with this were found to be women’s age 0.73 AOR [95% C.I 0.6–0.9], being lower wealth 0.22 AOR [95% C.I 0.07–0.6] as compared to lowest, parity 2.1 AOR [95% C.I 1.4–2.9], number of children at first use of contraceptive 1.1 AOR [95% C.I 1.03–1.19], having final say with provider 0.03 AOR [95% C.I 0.003–0.23] as compared with own decision making.

**Conclusion:**

Unmet need of family planning in Ethiopia was generally high especially with significant disparity in residence and regional states. Socio-demographic factors (age and wealth status) and obstetric factor (parity) were found to be significant factor. Informed decision making for provision of contraceptives and enhancing women’s awareness starting their childbearing life with family planning were recommended.

## Plain English summary

This study examines the multiple predictors of unmet need for family planning. Through structured interviews of women in the reproductive age group in Ethiopia, the study seeks to determine socio-demographic, socio-economic and reproductive health factors how much eligible women for contraception received family planning to meet their need. The study finds more than 16 % of the women surveyed were unmet need for family planning. The factors that most predict this were found to be the age of women, household wealth status, parity, number of children at first use of contraceptive and having final say with provider to use contraceptive. We believe that our study makes a significant contribution to the literature because it provides a multivariate analysis of the complex set of factors whose predict meeting the need of family planning has been studied in an isolated fashion thus far. This study is also significant as it provides cogent policy recommendations for the design and implementation of economic and non-economic interventions into realize women’s need for family planning.

## Article summary

### Strength of the study

The study was a secondary analysis of population level data about sociodemographic and belief/behavioral factors associated with unmet need for family planning. In their multivariable regression, they noted that unmet need was associated with age, number of living children at first use of family planning, wealth quintile, decisional autonomy, and parity.

### Limitations of the study

Being a survey, it is difficult to establish cause effect relationship.

## Introduction

Unmet need to family planning means when a married women who are able to give birth and want to stop or delay childbearing but are not using any method of contraception to prevent pregnancy [1]. One in ten married women face unmet need for family planning worldwide whereas, as many as one in five women in Africa. In 2017 met need of family planning among married or in-union women of reproductive age was 78% Worldwide. There is a difference in met need across different regions is lowest in Africa as 56% and above 75% in all other regions [2].

In Ethiopia despite family planning (FP) interventions of the Ethiopian federal ministry of health (EFMOH), including the Health Extension Program have significantly improved access to FP services [3], 22% of currently married women have an unmet need for family planning according to Ethiopian demographic health survey (EDHS) 2016 [4].

Unmet need for family planning is a valuable concept that is widely used for advocacy, the development of family planning policies, and the implementation and monitoring of family planning programs worldwide [5].

In each country, understanding the size of unmet need and the characteristics of women with unmet need can help planners strengthen programs. Survey data on unmet need can provide overall direction by helping to pinpoint the obstacles in society and weaknesses in services that need to be overcome [6].

Contraceptive utilization rate is a good indicator of the extent to which couples have access to reproductive health services [7]. The gap between women’s reproductive intentions and their contraceptive behavior can be revealed by unmet need for family planning. The indicator is useful for tracking progress towards the target of achieving universal access to reproductive health. Information on contraceptive prevalence complements the indicator of unmet need for family planning [8]. The odds of unintended pregnancy were about 16 fold among women who reported facing unmet need for contraception compared to those who did not [9].

The projected unmet need for family planning is to remain above 10% worldwide up to 2030 despite the reductions anticipated for some regions. The largest declines are expected in Eastern Africa, where unmet need is projected to fall from 22% in 2017 to 16% in 2030. Living up to the commitment of the international community to achieve universal access to reproductive health by 2030 will require intensified support for family planning, including through the implementation of effective government policies and program. Access to health care services and the realization of reproductive rights for all people will be essential to fulfil the pledge of the 2030 Agenda for Sustainable Development that “no one will be left behind” [2].

Different literatures agree that different factors influencing unmet need of family planning were older age, high-parity, non-Muslim women to have an unmet need to limit fertility [10]. Knowledge of contraceptive method and discussion with partner and health extension workers [11]. A visit to a health facility, exposure to family planning from media, and an educational difference between a husband and wife Rural residence [12], contraception related factors like availability, accessibility, affordability, side effects as a cause for unmet need [13], partner attitude towards family planning services utilization, current menstrual status, healthcare providers visit and discussion about family planning issues [14], high for young married women in the richest wealth quintile and among unmarried women [15], Women who were housewife/farmers were more likely to have unmet need than those employed women [16]. Number of living children, ever use of contraceptive methods [17]*,* social pressures, and provider bias limit knowledge about available options and access to appropriate methods, leading to higher rates of contraceptive failure and discontinuation after short periods. Addressing these barriers will improve maternal and child health, increase educational attainment and improve economic opportunities for young women [18].

The major reasons for not using contraceptives was inaccessibility of family planning methods, lack of knowledge, religious belief, fear of side effects of contraceptives they had suffered in the past, and opposition by husband [19], discontinuation due to health concerns and pressure from the surroundings were the most common cited reasons for non-use [20], less perceived risk of pregnancy due to breast feeding, religious prohibition [16].

## Methods and materials

**Population** : All women of reproductive age women in Ethiopia.

This is an analysis of cross-sectional data from the performance, monitoring and accountability (PMA)2020, obtained via phone survey of a representative sample of women in Ethiopia, divided into enumeration areas, with responses weighted according to Central Statistical Authority (CSA) probabilities to ensure generalizability.

### Sample design

PMA2020 Ethiopia uses a two-stage cluster design with residential area (urban and rural) and sub-regions as strata, sampling across all 11 geographic regions in Ethiopia. 95% of the target population, women of reproductive age 15–49 years, reside in five regions (Addis Ababa, Amhara, Oromia, SNNP and Tigray). Other regions with a total of less than 5% of the target population are allocated to a sixth synthetic region (referred to as “other”). Given the uneven population distribution and resource limitation, regional representative samples are only taken in the five regions (Addis Ababa, Amhara, Oromia, SNNP and Tigray). The fourth round sample of 221 EAs and 7651 households is designed to estimate modern contraceptive prevalence among all women at less than 2% margin of error at the national level, less than 3% for urban and rural estimates, and less than 5% at each of the five regional levels.

### Questionnaires

The PMA2020 is a comprehensive questionnaire that includes among other subjects, family planning. The field supervisors themselves administered the service delivery points(SDP) questionnaire at an additional three public SDPs that serve each enumeration areas(EA); the lowest, second lowest and third lowest-level public health SDPs (health post, health center, and district hospital) designated to serve each EA population.

Once listed, 35 households were randomly selected by field supervisors using a phone-based random number -generating application. All occupants in selected households were enumerated and from this list, all eligible women age 15–49 were approached and asked to give informed consent to participate in the study.

PMA2020 used standardized questionnaires for households, females and service delivery points (SDPs) to gather data about households, individual females and SDPs that are comparable across program countries and consistent with existing national surveys. Prior to launching the survey in each country, public health experts from Addis Ababa University review and modify these questionnaires to ensure all questions are appropriate to each setting. The questionnaires were translated into three local languages.

The household questionnaire gathers basic information about the household, such as ownership of durable goods, as well as characteristics of the dwelling unit, including wall, floor, and roof material, water sources and sanitation facilities. This information is used to construct a wealth quintile. The first section of the household questionnaire, the household roster, lists basic demographic information about all usual members of the household and visitors who stayed with the household the night before the interview. This roster is used to identify eligible respondents for the female questionnaire. In addition to the roster, the household questionnaire also gathers data that are used to measure key water, sanitation and hygiene (WASH) indicators, including regular sources and uses of WASH facilities and prevalence of open defecation by household members. The female questionnaire is used to collect information from all women age 15 to 49 who were listed on the household roster at selected households. The female questionnaire gathers specific information on education; fertility and fertility preferences; family planning access, choice and use; quality of family planning services; exposure to family planning messaging in the media; and the burden of collection water on women. The SDP questionnaire is used to collect information about the provision and quality of reproductive health services and products, integration of health services, and water and sanitation within the SDP.

### Data collection and processing

Fieldwork training started with a two-week training of new 30 new field staff, followed by a three-day refresher training for returning field staff before actual data collection. Data was collected from March to April 2016. Unlike traditional paper and-pencil surveys, PMA2020 uses Open Data Kit (ODK) Collect, an open-source software application, to collect data on mobile phones. All the questionnaires were programmed using this software and installed onto all project smartphones. The ODK questionnaire forms are programmed with automatic skip-patterns and built-in response constraints to reduce data entry errors.

This instantaneous aggregation of data also allowed for concurrent data processing and course corrections while PMA2020 was still active in the field. Throughout data collection, central staff at Addis Ababa University in Ethiopia and the data manager from the Gates Institute in Baltimore, Maryland routinely monitored the incoming data and notified field staff of any potential errors, missing data or problems found with form submissions on the central server. The use of mobile phones combined data collection and data entry into one step; therefore, data entry was completed when the last interview form was uploaded at the end of data collection in June, 2017. Once all data were on the server, data analysts cleaned and de-identified the data, applied survey weights, and prepared the final data set for analysis using Stata® version 14 software.

### Measurement

Women’s unmet need for contraception were measured by asking questions all eligible women (all non-pregnant women’s of reproductive age group) whether they are currently using any form of contraceptives or not. Those currently not using any form of contraceptive further asked whether they do not wish to become pregnant at all (unmet need for limiting) or within the next two years (unmet need for spacing). Finally, Percentage of fertile, sexually active women ages 15–49 who are not using contraception both for spacing and limiting were computed to get unmet need for contraceptives.

### Statistical analysis

Stata® version 13.0 were used to for analysis. The data were also explored again for inconsistencies and missing values. Variables having *p*-value less than 0.25 in binary logistic regression and variables of interest by investigators were selected as candidate for multivariable logistic regression. Multi-collinearities were checked among candidate variables if they have linear association among each other before entering them into multivariate logistic regression model for. Backward stepwise regression method was run by using entering at 0.1 and removal at 0.05. Finally, Odds ratio with 95% confidence interval and *P*-value less than 0.05 computed to assess the presence and degree of association and statistical significance independent predictors. The model fitness was checked using Hosmer and lameshaw test.

### Operational definitions

#### Unmet need for family planning

Proportion of women who [1] are not pregnant and not postpartum amenorrhoeic and are considered fecund and want to postpone their next birth for 2 or more years or stop childbearing altogether but are not using a contraceptive method, or [2] have a mistimed or unwanted current pregnancy, or [3] are postpartum amenorrhoeic and their last birth in the last 2 years was mistimed or unwanted Percentage of fertile, sexually active women aged 15–49 who are not using contraception and do not wish to become pregnant at all (unmet need for limiting) or within the next two years (unmet need for spacing) [4].

#### Utilization of family planning

Use of modern family planning method to prevent or postpone pregnancy.

#### Reasons for non-use

Reasons for non-use of contraceptive methods among married women who express a desire to postpone their next birth by two or more years.

#### Method chosen by self or jointly

Percentage of women ages 15–49 currently using a modern contraceptive method, reporting they decided on method themselves or jointly with a partner or provider.

#### Number of living children at first contraceptive use

Average number of living children at first contraceptive use among women ages 15–49 who have ever used contraception.

## Result

### Socio-demographic characteristics of women of reproductive age in Ethiopia, 2017

Seven thousand four hundred and ninety-four all women of reproductive age were interviewed yield response rate of 99.2%. The mean (+ standard deviation) age of the women was 27.8 (+ 0.14). Four thousand six hundred seventy-one (62.3%) of the women were married. Three thousand one hundred eighteen (41.6%) of the respondent never attended formal education. Two thousand four hundred and sixty-one (32.8%) of the women were nulliparous see Table 1.

**Table 1:** Socio demographic characteristics of women of reproductive age in Ethiopia, 2017

Variables	categories	Weighted Percentage of sample	Weighted number	Unweighted number
Age	15–19	23.6	1771	1735
	20–24	17.5	1308	1449
	25–29	18.2	1368	1447
	30–34	14.0	1053	1008
	35–39	11.6	871	824
	40–44	8.2	612	562
	45–49	6.8	512	456
Marital status	Married	62.3	4671	4224
	Living together	1.5	112	122
	Divorced	6.9	515	590
	Widowed	2.7	200	190
	Never married	26.6	1994	2351
Parity	None^a^	32.8	2461	2827
	1–2	23.8	1784	2019
	3–4	16.1	1209	1151
	5 or more	27.2	2039	1480
Education	Never attended	41.6	3118	2443
	Primary	38.5	2885	2692
	Secondary	15.6	1168	1683
	Technical/Vocational	2.7	202	393
	University	1.5	116	26
Wealth quintile	Lowest	19.3	1449	923
	Lower	19.3	1445	963
	Middle	19.2	1440	972
	Higher	19.6	1471	1253
	Highest	22.6	1690	3370
Region	Addis	5.0	378	891
	Amhara	22.7	1702	1285
	Oromiya	38.4	2877	1736
	SNNP	22.4	1681	1594
	Tigray	6.7	499	1134
	Other	4.8	357	841
	**Total**	**100.0**	**7494**	**7481**

^a^stands for those never gave birth

### All women of reproductive age unmet need for family planning, in Ethiopia

**Fig. 1:**
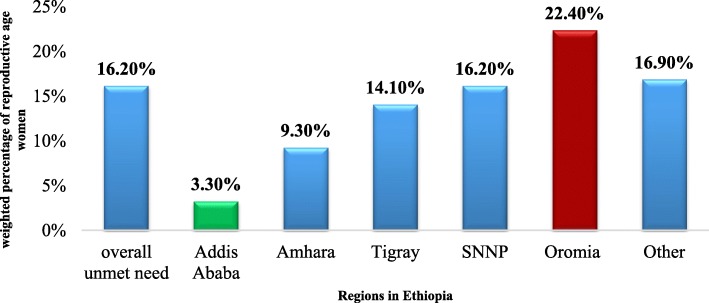
Regional status of all woman of reproductive age Unmet Need for family planning, in Ethiopia, 2017

Overall unmet need for family planning among all women was 1214 (16.2%) of which 772 (10.3%) was for spacing and 450 (6.0%) for limiting while 1799 (24.0%) among married women. It was 540 (7.2%) in urban and 1431(19.1%) in rural areas of Ethiopia. There is a regional disparity which is 22.4% in Oromia region while only 3.3% in Addis Ababa of all women have unmet need to family planning see Fig. 1.

### Reasons for not using family planning in Ethiopia

**Fig. 2:**
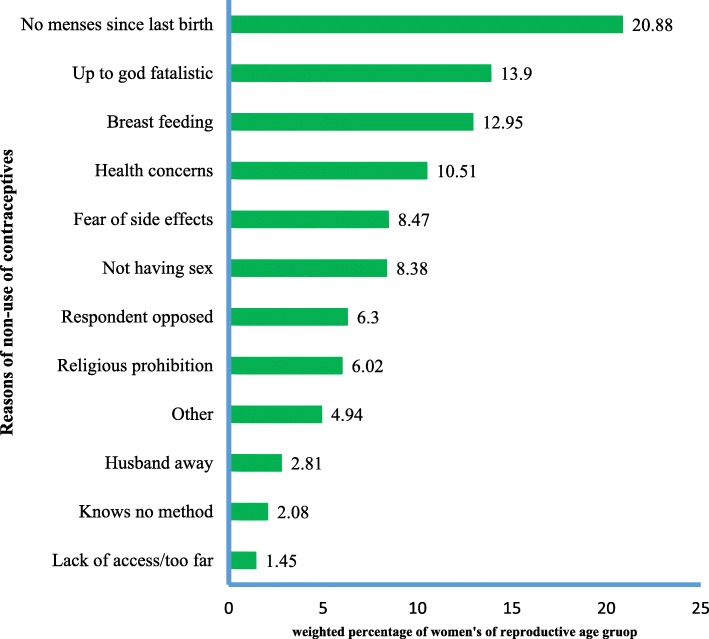
Reasons for non-use of contraception among women’s of reproductive age group in Ethiopia, 2017

The most common reason for non-use of contraceptives among married women were four hundred sixty-one (20.88%) no menses since last birth, three hundred seven (13.90%) women says it is up to god and/or fatalistic, two hundred eighty-six (12.95%), two hundred thirty-two (10.51%) were health concerns, one hundred eighty-seven (8.47%) fear of side effects and one hundred eighty-five (8.38%) not having sex see Fig. 2.

### Predictors of unmet need for family planning in Ethiopia in 2107

Multivariate logistic regression model revealed that being younger age, having a final say jointly with family planning provider, having less number of living children at first use of contraceptive mothers, being in second wealth quintile as compared with first wealth quintile, having less parity were found to be significant predictors unmet need for contraception.

After controlling for potential confounders in the multivariate analysis revealed that as age of women’s increase by one year the odds of being unmet need for family planning 20% less likely (AOR 0.8 [95% C.I 0.75–0.9]), having a final say jointly with family planning provider were 96% less likely as compared women’s own final decision to be unmet need of family planning (AOR 0.04 [95% C.I 0.005–0.300]), as the number of living children at first use of contraceptive increases by one the odds being unmet for family planning 1.1 more likely (AOR 1.1 [95% C.I 1.01–1.2]), mothers in lower wealth quintile were 80% (AOR 0.2 [95% C.I 0.06–0.60]) less likely to be unmet need for family planning as compared to those in lowest wealth quintile, As parity increase with one birth the odds of being unmet need for family planning were twice more likely (AOR 2.1 [95% C.I 1.4–2.8]) see Table 2.

**Table 2:** Multivariate logistic regression model of unmet need for family planning in Ethiopia in 2017

Variables	Unmet need for family planning		COR (CI 95%)	AOR (CI 95%)
	Yes (%)	No (%)		
Age^a^	1217(16.2)	6277(83.8)	1.02(1.01–1.03)**	0.80(0.75–0.90)**
Final decision				
You alone	70(43.5)	930 (39.8)	1.0	1.0
Provider	0.21(0.1)	63(2.7)	0.04 (0.01–0.33)*	0.08 (0.006–1.2)
Partner	15(9.3)	222(9.5)	0.86(0.36–2.01)	1.20(0.30–4.0)
You and provider	5(3.1)	110(4.7)	0.60(0.09–9.95)	0.04(0.01–0.26)**
You and partner	71(44.1)	1009(43.2)	0.93(0.60–1.40)	1.98(0.95–4.12)
Number of children at first use ^a^	1217(16.2)	6277(83.8)	1.05(1.01–1.09)*	1.10(1.01–1.20)*
Wealth quintile				
Lowest	349(28.6)	1099 (17.5)	1.0	1.0
Lower	284(23.3)	1161(18.5)	0.76 (0.59–0.98)*	0.20(0.06–0.58)**
Middle	268(22.0)	1172(18.6)	0.72 (0.55–0.92)*	0.580(0.20–1.89)
Higher	203(16.7)	1268(20.2)	0.50 (0.38–0.65)**	0.57(.20–1.90)
Highest	113(9.3)	1577(25.1)	0.22 (0.17–0.29)**	0.89(0.18–4.20)
Parity ^a^	1217(16.2)	6277(83.8)	1.92(1.79–2.06)**	2.10(1.39–2.78)**

^a^Continuous variable,*Statistically significant at *P*-value < 0.05, ** statistically significant at *P*-value < 0.001, *COR* crude odds ratio, *AOR* adjusted odds ratio, 1.0 reference category. *NB* frequencies were weighted

## Discussion

The gap between women’s reproductive intentions and their contraceptive behavior can be revealed by unmet need for family planning. Unmet need for contraception is useful for tracking progress towards the target of achieving universal access to reproductive health. The level of unmet need of family planning needs to be assessed periodically and identifying effective intervention areas has of great implication.

This study shows that unmet need of family planning in Ethiopia was among all women was 16.2%. It shows a little increment as compared with Ethiopian demographic health surveys (EDHS) in 2016 which is 15.2% among all women. This may be due to changes seen within a year for different levels of interventions and EDHS data a five year history while PMA2020 data collected annually [4].

This study also showed there were disparities among different regions of Ethiopia for instance unmet need of family planning among reproductive age married women in Addis Ababa were 6.9, 13.4% in Amhara region, 31.0% in Oromia region and 24.0% in southern nations, nationalities and peoples (SNNP) of Ethiopia. A cross sectional study in Enemay District, in 2013 and West Belessa district in 2015 both North western Ethiopia showed 25.6 and 39.5% unmet need for contraceptives respectively, which were higher as compared to this study finding in Amhara region. This shows that unmet need for contraception varies from place to place with sociocultural diversity, differentials in geographic and economic accessibility of reproductive health services for the women’s [14, 17].

The main reasons for unmet need for contraceptives were found to be no menses since last birth, it is up to god and/or fatalistic, health concerns, fear of side effects and not having sex. This were supported with different cross-sectional studies in Saudi Arabia in 2018 found fear of side effect, in Egypt in 2017 discontinuation due to health concerns and in Dangila town, Awi Zone, Amhara regional state of Ethiopia in 2015 identified religious prohibition as main reasons for non-use of contraception [16, 19, 20].

In this study age of women were statistically significant that an increase by one year of age of the women, the odds of being unmet need for family planning would be 20% less likely. Similarly a demographic health survey comparative reports 34 in developing countries in 2014 reveals that unmet need to family planning was higher among younger women (age 15–19) than older women (age 20–24) and also another cross sectional study in 2015 in West Belessa District, North Western Ethiopia, among married women with 35 years and above were 70% less likely to have unmet need than women with 15–19 age group [14, 15]. This might be younger age groups have no adequate information regarding contraception and comprehensive education of sexuality and also majority of Ethiopians reside in rural areas in which contraceptive use among adolescent age groups believed to be shameful.

The study found that mothers in lower wealth quintile were 80% less likely to be unmet need for family planning as compared to those in lowest wealth quintile. Likewise, a comparative reports of the demographic health survey in 34 of developing countries in 2014 showed high unmet need for contraception among young married women in the richest wealth quintile [14]. This indicates that being in high wealth index doesn’t guarantee for met need for family planning.

Parity was also found to be significant predictor of unmet need for contraception. As parity increase with one birth the odds of being unmet need for family planning were twice more likely. West Belessa district in 2015, revealed that, the odds of having unmet need for contraception increase as the number of living children increases [17]. This shows that having children in Ethiopia is unplanned and that is why maternal mortality and morbidity were high in the country. There would be no planned family can be without met-need of contraception.

The study revealed that having a final say jointly with family planning provider were 96% less likely as compared women’s own final decision to be unmet need of family planning. This finding was supported with a cross sectional study in Misha district, Southern Ethiopia, in 2014 which revealed that having knowledge of contraceptive method and discussion with health extension workers were helpful for met need [11]. Decision regarding contraception was better if made with provider as informed decision was helpful for continuous use of contraceptive method of their choice as different women have different health problems and reproductive health goals choice of contraceptive individually. This study utilized country level data which is more representative and up to date than demographic health survey as the data were collected on annual bases. Being a survey, it is difficult to establish cause effect relationship.

## Conclusion

More than 16 % of women were responded that their need for contraception still not met. There was also great disparity regarding unmet need for family planning among urban and rural and also a regionally. The major reason for non-use of contraception were found to be no menses since last birth, it is up to god and/or fatalistic, health concerns, fear of side effects and not having sex.

Having a final say jointly with family planning provider, being younger age, having less number of living children at first use of contraceptive mothers, being in second wealth quintile as compared with first wealth quintile, having less parity were found to be significant predictors unmet need for family planning.

## Recommendation

- The disparities between urban and rural difference in unmet need for contraception regional difference should resolved strengthening the health systems and more advocacy through different information, education and communication making an agenda.
- Public health interventions on meeting the need for contraception among women should promote women to make informed decision regarding contraceptive use. Priorities should be given for younger age, those in second wealth quintile, having more child before starting contraception use and multi-parous women.

## Abbreviations

CSA: Central statistical agency
EA: Enumeration areas
EDHS: Ethiopian demographic health survey
EFMOH: Ethiopian federal ministry of health
FP: Family planning
ODK: Open Data Kit
PMA: Performance monitoring and accountability
SDPs: Service delivery points
